# Retraction: The effect of personality traits on employees' annual salaries in Chinese startups

**DOI:** 10.3389/fpsyg.2023.1268868

**Published:** 2023-08-03

**Authors:** Frontiers Editorial Office

Following publication, concerns were raised regarding the methodological soundness and scientific validity of the article. An investigation was conducted in accordance with Frontiers' policies. The author failed to provide a satisfactory explanation and as a result, the conclusions of the article have been deemed unreliable and the article has been retracted.

The retraction was approved by the Specialty Chief Editor of Personality and Social Psychology. The author agreed to this retraction.

